# Real-World Rates of Bleeding, Factor VIII Use, and Quality of Life in Individuals with Severe Haemophilia A Receiving Prophylaxis in a Prospective, Noninterventional Study

**DOI:** 10.3390/jcm10245959

**Published:** 2021-12-18

**Authors:** Gili Kenet, Yeu-Chin Chen, Gillian Lowe, Charles Percy, Huyen Tran, Annette von Drygalski, Marc Trossaërt, Mark Reding, Johannes Oldenburg, Maria Eva Mingot-Castellano, Young-Shil Park, Flora Peyvandi, Margareth C. Ozelo, Johnny Mahlangu, Jennifer Quinn, Mei Huang, Divya B. Reddy, Benjamin Kim

**Affiliations:** 1 The National Hemophilia Center, Amalia Biron Research Institute of Thrombosis and Hemostasis, Sheba Medical Center, Tel Aviv University, Tel Hashomer, Tel Aviv-Yafo 52621, Israel; 2 Haemophilia Care and Research Center, Tri-Service General Hospital, National Defense Medical Center, Taipei 11490, Taiwan; yeuchin99@gmail.com; 3 West Midlands Comprehensive Care Haemophilia Centre, Queen Elizabeth Hospital, Birmingham B15 2TH, UK; gillian.lowe@uhb.nhs.uk (G.L.); charles.percy@uhb.nhs.uk (C.P.); 4 Haemophilia Treatment Centre, Haemostasis & Thrombosis Unit, The Alfred Hospital, Melbourne, VIC 3004, Australia; huyen.tran@monash.edu; 5 Department of Molecular Medicine, Scripps Research Institute, La Jolla, CA 92037, USA; avondrygalski@ucsd.edu; 6 Centre Régional de Traitement des Hémophiles, CHU de Nantes, 44093 Nantes, France; marc.trossaert@chu-nantes.fr; 7 Center for Bleeding and Clotting Disorders, University of Minnesota, Minneapolis, MN 55454, USA; redin002@umn.edu; 8 Center for Rare Diseases and Institute of Experimental Haematology and Transfusion Medicine, University Hospital Bonn, 53127 Bonn, Germany; johannes.oldenburg@ukbonn.de; 9 Regional University Hospital, Carlos Haya, 29010 Málaga, Spain; memingot@gmail.com; 10 Kyung Hee University Hospital at Gangdong, Seoul 134-727, Korea; pysped@khu.ac.kr; 11 Fondazione IRCCS Ca’ Granda Ospedale Maggiore Policlinico, Angelo Bianchi Bonomi Hemophilia and Thrombosis Center and Fondazione Luigi Villa, 20122 Milan, Italy; flora.peyvandi@unimi.it; 12 Department of Pathophysiology and Transplantation, Università degli Studi di Milano, 20122 Milan, Italy; 13 Hemocentro UNICAMP, Department of Internal Medicine, School of Medical Sciences, University of Campinas, Campinas 13083-878, SP, Brazil; margaret@unicamp.br; 14 Hemophilia Comprehensive Care Center, Charlotte Maxeke Johannesburg Academic Hospital, University of the Witwatersrand and NHLS, Johannesburg 2193, South Africa; johnny.mahlangu@nhls.ac.za; 15 BioMarin Pharmaceutical UK Ltd., London WC1A 2SL, UK; jennifer.quinn@bmrn.com; 16 BioMarin Pharmaceutical Inc., Novato, CA 94949, USA; Mei.Huang@bmrn.com (M.H.); divya.reddy@bmrn.com (D.B.R.); ben.kim@bmrn.com (B.K.)

**Keywords:** haemophilia A, noninterventional study, FVIII prophylaxis

## Abstract

Regular prophylaxis with exogenous factor VIII (FVIII) is recommended for individuals with severe haemophilia A (HA), but standardised data are scarce. Here, we report real-world data from a global cohort. Participants were men ≥18 years old with severe HA (FVIII ≤ 1 IU/dL) receiving regular prophylaxis with FVIII. Participants provided 6 months of retrospective data and were prospectively followed for up to 12 months. Annualised bleeding rate (ABR) and FVIII utilisation and infusion rates were calculated. Differences between geographic regions were explored. Of 294 enrolled participants, 225 (76.5%) completed ≥6 months of prospective follow-up. Pre-baseline and on-study, the median (range) ABR values for treated bleeds were 2.00 (0–86.0) and 1.85 (0–37.8), respectively; the median (range) annualised FVIII utilisation rates were 3629.0 (1008.5–13541.7) and 3708.0 (1311.0–14633.4) IU/kg/year, respectively; and the median (range) annualised FVIII infusion rates were 120.0 (52.0–364.0) and 122.4 (38.0–363.8) infusions/year, respectively. The median (range) Haemo-QoL-A Total Score was 76.3 (9.4–100.0) (*n* = 289), ranging from 85.1 in Australia to 67.7 in South America. Physical Functioning was the most impacted Haemo-QoL-A domain in 4/6 geographic regions. Despite differences among sites, participants reported bleeding requiring treatment and impaired physical functioning. These real-world data illustrate shortcomings associated with FVIII prophylaxis for this global cohort of individuals with severe HA.

## 1. Introduction

Haemophilia A (HA) is an X-linked recessive bleeding disorder caused by mutations in the gene that codes for factor VIII (FVIII) protein, an essential cofactor in the coagulation pathway. The estimated prevalence of HA is 17.1 per 100,000 males, with about one-third affected by severe HA, defined as FVIII activity < 1 IU/dL [1,2]. Clinical manifestations of severe HA include frequent spontaneous bleeding episodes, predominantly in joints and soft tissues, which can lead to debilitating multiple-joint arthropathies and substantially increased risk of death [3]. Regular prophylaxis is recommended for individuals with severe HA [3]. Although recent advances in haemophilia treatment and management have dramatically increased quality of life (QoL), individuals with severe HA still experience bleeding events [4], indicating an unfulfilled need for greater haemostatic control in this population. These unmet needs in those receiving prophylactic FVIII replacement therapy are incompletely characterised on an individual and population level [5,6]. Standardised data on bleeding, FVIII use, and QoL in the setting of routine clinical practice for patients on prophylaxis are needed to better understand the burden of both severe HA and the prophylactic treatment approach.

This multinational, prospective, noninterventional study collected standardised real-world data on bleeding episodes, haemophilia medication use, and health-related QoL from a global, heterogeneous population of participants with severe HA on currently available FVIII prophylaxis. This study was also a run-in for the sponsor’s phase 3 gene therapy studies (Clinicaltrials.gov NCT03370913/EudraCT 2017-003215-19, NCT03392974/EudraCT 2017-003573-34).

## 2. Materials and Methods

### 2.1. Study Design and Participants

This prospective, multicentre, multinational, noninterventional, longitudinal study was conducted in compliance with local regulations, International Conference on Harmonisation Guidelines for Good Clinical Practice, and the Declaration of Helsinki; ethics committees or review boards at all participating sites approved the protocol (Supplementary S1). Participating sites were located in Australia, Belgium, Brazil, France, Germany, Israel, Italy, South Africa, South Korea, Spain, Taiwan, the UK, and the US. Sites were selected based on selective global outreach to haemophilia treatment centres that expressed interest in participating in a future gene therapy trial and were deemed to be capable of doing so. Participants were males ≥18 years old with severe HA (FVIII activity ≤ 1 IU/dL) continuously treated with prophylactic exogenous FVIII for ≥6 months [2].

Planned enrolment was up to 250 participants followed for ≥6 months and ≤12 months. After 6 months, potentially eligible participants could be screened for entry into a phase 3 interventional study of valoctocogene roxaparvovec (Clinicaltrials.gov NCT03370913/EudraCT 2017-003215-19, NCT03392974/EudraCT 2017-003573-34). To be eligible, participants had to have received prophylactic FVIII therapy for ≥6 months prior to study entry and been treated/exposed to FVIII concentrates or cryoprecipitate for ≥150 exposure days. Participants could not have a history of detectable FVIII inhibitors. After being initially eligible for enrolment, participants positive for human immunodeficiency virus (HIV) infection were excluded by a protocol amendment—secondary to the development of elevated liver enzyme levels by an HIV-positive participant who was receiving a combination of highly active antiretroviral therapy (HAART) and valoctocogene roxaparvovec—out of caution for the long-term liver health of HIV-positive participants receiving HAART who may be interested in receiving gene therapy. Additional exclusion criteria, including significant liver dysfunction, chronic or active hepatitis B, and active hepatitis C, are described in detail in (Supplementary S2). High-quality historical documentation concerning bleeding and exogenous FVIII usage over the previous 6 months was required. Retrospectively collected pre-baseline data were compared to and combined with data collected during the study period. There were no minimum requirements of annualised bleeding rates, either before or during this study, that could impact participants’ eligibility for the future gene therapy trial. However, the possibility of future enrolment in gene therapy a trial may have been a motivating factor for some participants to enrol in this study.

Pre-existing host humoral and cellular immunity against the AAV capsid are known to negatively affect the efficacy of AAV-vector-based gene therapies [7]; thus, participants were also screened for pre-existing adeno-associated virus 5 (AAV5). Although antibody status did not impact eligibility for this trial, participants positive for anti-AAV5 antibodies were not eligible for enrolment in the subsequent gene therapy trial.

Study procedures included the review and entry of bleeding episode and haemophilia medication data on at least a monthly basis, as well as the collection of concomitant medications, adverse events (AEs), serious AEs (SAEs), and interim medical history at each visit or during telephone calls on at least a monthly basis. Except for screening/baseline and end-of-study visits, all other study visits occurred according to participants’ local standard of care. No clinical intervention or study drug was provided.

### 2.2. Study Assessments and Endpoints

After enrolment, participants reported bleeding episodes and haemophilia medication data on a weekly basis for the duration of the study. Details of each bleeding episode were captured, including start date/time, type (e.g., joint or muscle), location, and whether there was preceding trauma or ensuing treatment. Specific reporting of all administered haemophilia medications was required, including start/date time, product name, dose, and reason for use (e.g., usual prophylaxis, one-time prophylaxis, or treatment for bleed). As the prescribed frequency of usual FVIII prophylaxis was not captured, the manual review of listings was required to adjudicate the prescribed frequency of infusions and assess adherence. The primary clinical endpoint was the annualised number of bleeding episodes (annualised bleeding rate (ABR)) requiring exogenous FVIII replacement treatment. Secondary clinical endpoints included the annualised utilisation (IU/kg/yr) and infusion rate (count/yr) of exogenous FVIII replacement therapy. Participants also completed 4 patient-reported outcome (PRO) assessments on day 1, including the haemophilia-specific health-related quality of life questionnaire for adults (Haemo-QoL-A) [8,9], EQ-5D-5L [10,11], Haemophilia Activities List (HAL) [12], and Work Productivity and Activity Impairment plus Classroom Impairment Questions: Haemophilia Specific (WPAI+CIQ:HS) [13,14], as described in Supplementary S2. Safety assessments consisted of monitoring AEs (coded using the Medical Dictionary for Regulatory Activities v20.1) and measuring vital signs and haematology, clinical chemistry, and urinalysis variables.

### 2.3. Statistical Analysis

Baseline and safety analyses included all enrolled participants. Bleeding and FVIII usage were analysed for those with ≥6 months of on-study data (6-Month Analysis Population). ABR was calculated using only treated bleeds for the primary analysis; bleeds due to surgery/procedure were not included. ABR was defined as number of bleeding episodes during the calculation period/total number of calculation period days × 365.25. ABR and FVIII usage rates were determined for the 6 months before day 1 (pre-baseline), 6 months after day 1 (on-study), and total duration (pre-baseline and on-study). Supportive analyses included all bleeds (treated and non-treated), joint bleeds, problem joint bleeds, spontaneous bleeds, and traumatic bleeds. Results were also evaluated by prophylaxis type (standard half-life (SHL), extended half-life (EHL), or plasma-derived (PD)) and geographic regions in which the sites were located to provide a more nuanced understanding of the data. The regional analysis included the following groups: Australia (6 sites), Europe/Middle East (3 sites in Belgium, 6 in France, 2 in Germany, 1 in Israel, 2 in Italy, 3 in Spain, and 9 in the UK), Africa (2 sites in South Africa), North America (16 sites in the US), South America (1 site in Brazil), and East Asia (3 sites in South Korea and 5 in Taiwan). All variables were descriptively summarised. As the trial was not designed to test a statistical hypothesis, power analyses to determine sample size were not performed.

## 3. Results

### 3.1. Baseline Characteristics

**Figure 1:**
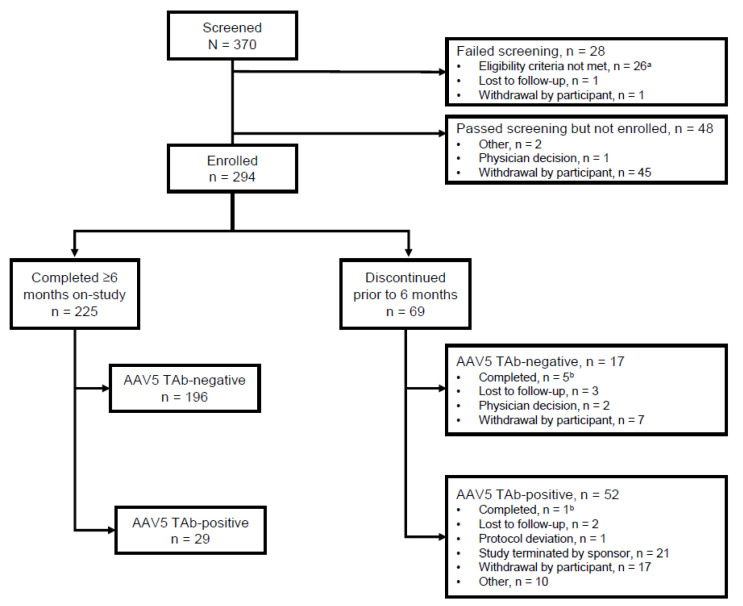
Patient disposition. ^a^ Reasons for screen failure: no history of FVIII inhibitor and results from a Bethesda assay, *n* = 8; significant liver dysfunction with abnormal laboratory results, *n* = 3; active hepatitis C, *n* = 6; chronic or active hepatitis B, *n* = 1; concurrent enrolment in another clinical study, *n* = 2; ability to comply with protocol requirements per Investigator, *n* = 2; male ≥18 years of age with residual FVIII ≤1, *n* = 1; must have been on prophylaxis FVIII for ≥6 months prior, *n* = 4. ^b^ Completing 6 months on-study was not required for participants not rolling over into the interventional study. AAV5 TAb, adeno-associated virus vector total antibody; FVIII, factor VIII.

Of the 370 participants who were screened, 28 did not meet screening criteria and an additional 48 (13%) passed screening but did not enrol (Figure 1). Of these 48 participants, 47 were positive for AAV5 antibodies at screening, thus precluding their participation in the subsequent phase 3 gene therapy trial and likely impacting their decision not to enrol. Of 294 enrolled participants, 225 (76.5%) completed ≥6 months on-study and were included in the 6-Month Analysis Population.

Median (range) age at enrolment was 31.0 (18.0–71.0) years, including 85.0% who were ≤50 years old and 95.6% who were <65 years old. All participants were male, and 62.9% were white (Table 1). Overall, 34.4% of participants reported ≥1 problem joint, defined as a joint with chronic joint pain, chronic synovitis, haemophilic arthropathy, limited motion, or recurrent bleeding [15,16,17]. At baseline, among all enrolled participants, 198 (67.3%) reported a history of musculoskeletal and connective tissue disorders, including 139 (47.3%) with haemophilic arthropathy. Knee arthroplasty was reported by 41 (13.9%) participants.

### 3.2. Clinical Endpoints

For the 6-Month Analysis Population, the median (range) follow-up time was 225.0 (169–469) days. In this observational study, FVIII treatment regimens were not assigned, and a wide spectrum of prophylactic models was adopted by each participating site. A high adherence to prescribed prophylaxis was observed, and pre-baseline and on-study adherence data were consistent. For the total study duration, median (range) adherence was 91.9% (27%–100%) of the derived prescribed frequency of their FVIII regimen. Adherence to >80% of prescribed frequency was seen for 72.4% of participants (163/225), while <60% adherence was seen for 7.1% of participants (16/225).

Overall, pre-baseline ABR data were consistent with on-study data. For the 6-Month Analysis Population, the median (range) ABR for treated bleeds for the total duration was 2.27 (0, 57.8); ABRs were 2.00 (0, 86.0) during pre-baseline and 1.85 (0, 37.8) on-study (Table 2). Results for treated bleeds by types of bleeds had a similar overall pattern to that of all treated bleeds. No bleeds were reported by 55 (24%) participants.

The median (range) annualised FVIII utilisation rate for the 6-Month Analysis Population was 3680.9 (1359.1–13938.1) IU/kg/year, and the median (range) annualised FVIII infusion rate was 122.3 (52.0–363.9) infusions/year across the total duration (Table 3). Annualised FVIII utilisation and infusion rates were similar during the pre-baseline and on-study periods. The greatest contributor to usage was usual prophylaxis.

### 3.3. Patient-Reported Outcomes

**Figure 2:**
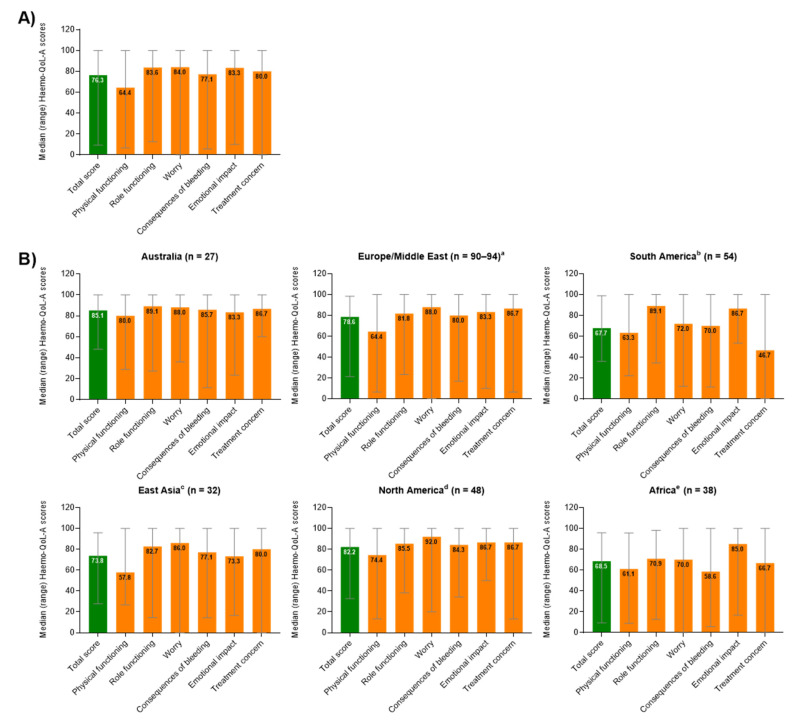
Median (range) overall transformed Haemo-QoL-A total and domain scores at baseline (**A**) for all participants globally (*n* = 298) and (**B**) for participants by region. Haemo-QoL-A scores range from 0 to 100, with higher scores indicating higher quality of life. ^a^ Belgium, Germany, Spain, France, UK, Israel, and Italy. For total score, *n* = 90; Physical functioning, *n* = 94; Role functioning, *n* = 94; Worry, *n* = 91; Consequences of bleeding, *n* = 93; Emotional impact, *n* = 91; Treatment concern, *n* = 94. ^b^ Brazil. ^c^ Korea and Taiwan. ^d^ US. ^e^ South Africa. Haemo-QoL-A, haemophilia-specific health-related quality of life questionnaire for adults; SD, standard deviation.

For 289/294 (98.3%) participants in the total population, the Haemo-QoL-A median (range) Total Score was 76.3, (9.4–100), with higher scores representing better health-related QoL (Figure 2A). The highest domain scores were observed for Emotional Impact, Role Functioning, and Worry, while the lowest scores were observed for Physical Functioning and Consequences of Bleeding.

The EQ-5D-5L index was completed by 264/294 (89.8%) participants, and the median (range) score was 0.767 (0.06–1.00). For all 294 enrolled participants, the median (range) score on the Visual Analog Scale (VAS) portion of the EQ-5D-5L was 80.0 (20.0–100.0). The median (range) HAL summary score for 293/294 (99.7%) participants was 80.5 (20.5–100.0) points, with higher scores representing better self-perceived functional abilities. For 289/294 (98.3%) participants, the median (range) WPAI+CIQ:HS Activity Impairment percentage score was 20.0% (0–90.0%), with higher percentages indicating greater impairment and less productivity. Most participants (286/294, 97.3%) completed their diary entries, with a median (range) of 28.5 (1.0–95.0) weeks of data collected. The median (range) percentage of missed days from work (calculated as missed days/expected workdays) for the total population was 0% (0–100%).

### 3.4. Comparative Analysis among Trial Sites by Region

At enrolment, the median age was lowest among participants in South America (27 years) and highest among participants from East Asia (40 years); the median weight was highest among participants from North America (77.5 kg), South America (78.1 kg), and Australia (79.0 kg) (Table 1). Rates of problem joints were highest among participants from East Asia (56.3%) and lowest among those from South America (9.3%) and Africa (18.4%). Exclusive EHL product use was highest among participants in Australia, followed by Europe/Middle East and North America (Figure S1). Plasma-derived products were primarily used by participants in Africa, while participants from the sites in East Asia generally either exclusively used SHL products or used a combination of FVIII product types during the study period (also including participants who switched products).

For most regions, pre-baseline and on-study ABR for treated bleeds were consistent (Table S1). A high degree of variability was noted in participants from East Asia during the pre-baseline period, but variation was lower during the on-study period. On-study median ABRs were highest in participants from the African sites (4.25) and lowest for participants at the South American (0.00) and Australian sites (1.68).

In all sites, FVIII utilisation rates were generally similar pre-baseline and on-study across FVIII products (Table S2). Participants in Africa reported the least FVIII use, followed by participants in South America and East Asia; participants in North America, followed by Australia and Europe/Middle East, reported the most.

On-study annualised infusion rates were consistent with pre-baseline values across regions (Table S3). Infusion rates for participants in North America were similar to those from sites in Australia and Europe/Middle East, with the highest infusion rates reported for participants at sites in South America.

Median Haemo-QoL-A total scores ranged from 85.1 for participants from sites in Australia to 67.7 for participants from sites in South America (Figure 2B). The most impacted domain was Physical Functioning in all regional groups except for South America and Africa. In South America, Treatment Concern (e.g., “I worry about the availability of haemophilia products”) had the lowest domain score, while in Africa, Consequences of Bleed (e.g., “I worry about accidents”) was the most impacted domain.

### 3.5. Safety

The incidence of AEs in the total population (*n* = 294) was 43.5%; no AEs led to study discontinuation (Table S4). The most common haemophilia-related AEs were arthralgia (5.8%), haemophilia arthropathy (2.0%), back pain (1.7%), and musculoskeletal pain (1.4%). SAEs were reported in 14 (4.8%) participants. No particular pattern of SAEs was noted; 5 SAEs were Grade 3 bleeding or haemophilia-related events, including haemorrhoidal haemorrhage 1 (0.3%), haemophilic arthropathy 1 (0.3%), oesophageal haemorrhage 1 (0.3%), haematuria 1 (0.3%), and haematoma 1 (0.3%).

## 4. Discussion

In this study, we present real-world data on bleeding, FVIII use, and QoL in a global cohort of participants with severe haemophilia A potentially interested in enrolling in a gene therapy trial. These data reflect a severe HA population receiving regular FVIII prophylaxis treatment. At baseline, a high proportion of participants reported joint-related comorbidities of haemophilia. Joint-related procedures, such as arthroplasty, were also common, confirming that this severe haemophilia population is more likely to have joint problems and need associated surgery at a younger age.

ABR and FVIII usage results in the pre-baseline period were generally similar to the on-study period, indicating reliability of retrospective data. For unknown reasons, a slight increase in treated traumatic bleeds on-study compared to pre-baseline was observed. However, this study was not powered for a statistical analysis comparing retrospectively and prospectively collected data. Additional research is needed to further evaluate the change in treated traumatic bleed rates observed here.

Our data are similar to previous reports of real-world bleeding and FVIII use in individuals with severe haemophilia A. In a global, noninterventional study that prospectively collected real-world data of 49 participants with severe HA treated with prophylactic FVIII, the mean (95% confidence interval) and median (Q1, Q3) ABRs were 5.0 (3.3, 7.5) and 1.9 (0.0, 0.82) for treated bleeds and 6.2 (4.2, 9.2) and 2.7 (0.0, 9.4) for all bleeds, respectively; the majority (83.7%) of participants received SHL prophylaxis [18]. Adherence to proscribed prophylaxis frequency was lower in that study than here, with 66.7% of participants reporting adherence to >80% of prescribed doses [18]. In another study enrolling 18 severe HA participants, the median baseline ABR for all bleeds was 7.5 (range: 4–28) and the median annualised FVIII utilisation was 3028 IU/kg/yr [19]. A large 12-month retrospective study involving 1346 males from seven European countries, mostly receiving regular prophylaxis (75% with severe HA) [4], reported comparable median (Q1, Q3) ABR values ranging from 1.0 (0, 2) to 4.0 (1, 12) for all bleeds and from 0.0 (0, 1) to 2.5 (1, 6) for joint bleeds. Similarly, an Austrian haemophilia registry study reported median (IQR) ABRs of 4.9 (1.6, 13.5) for all bleeding events and 4.1 (0.9, 12.3) for joint bleeding in 26 participants with severe HA receiving FVIII prophylaxis; the median (Q1, Q3) annualised FVIII utilisation was 3364 (2219, 4297) IU/kg/yr [20]. Overall, our data combined with these previous results suggest that breakthrough bleeding events requiring additional treatment occur for many individuals with severe HA receiving FVIII prophylaxis.

The QoL and PRO results from this study indicate substantial burden and impairments in this population despite good adherence to a variety of prophylactic treatment regimens. Health-related QoL was particularly impacted in the Physical Functioning domain, reflecting the residual burden of disease. The median Haemo-QoL-A total scores (76.3) in our study tended to be lower (indicating worse QoL) than those previously reported in small US (*n* = 21; mean, 85.6) [21] and Canadian (*n* = 33; mean, 85.8) [22] cohorts (100% and 93% with severe HA, respectively) but similar to those reported in an international cohort (*n* = 221; mean, 73.1; 52% with severe HA) [8], a discrepancy most likely due to the younger age and fewer comorbidities observed in the US and Canadian cohorts [8,22]. However, our median baseline EQ-5D and EQ VAS scores (0.767 and 80.0, respectively) were consistent with those from a single-centre UK study enrolling 44 patients with severe HA (mean (range) EQ-5D-5L, 0.68 (0.09–1) and HAL, 71.7 (30–100) scores) [23]. Importantly, the EQ-5D-5L may underestimate QoL burden in haemophilia due to the disability paradox reported in this population [24]. Lastly, perceived level of overall impairment due to HA as measured with the WPAI+CIQ:HS was rated as 30% in an observational multicentre study conducted in Italy (84 patients, 86% with severe HA) [25], slightly higher than that found in our study (24%).

When comparing trial sites grouped by geographic region, ABRs were highest at sites in East Asia and in Africa; these sites were also among those with the lowest FVIII utilisation in this study. Though Africa and East Asia comprise 39% of the total population of all countries participating in the WFH global survey, they receive 4% of exogenous FVIII products [26], making the recommended implementation of prophylaxis difficult [27]. In our study, participants in East Asia and Africa reported much lower infusion rates with SHL and/or plasma-derived FVIII than elsewhere, consistent with limited FVIII availability in these regions [26]. Low FVIII utilisation was also reported at sites in South America, where limited FVIII supply [26,28] may be partially mitigated by giving more frequent infusions, as the infusion rate there was the highest of any region in our study. The ABRs for participants in South America were similar to those reported here for developed regions. Interestingly, participants in Africa also reported low problem joint numbers but high ABRs. The comparatively low proportion of participants with problem joints in Africa and South America was unexpected given the importance of early, consistent prophylaxis for reducing joint damage [3,29] and limited and/or recent access to prophylaxis in these regions [28,30,31]. Problem joints may have been underreported at these sites. Further study is needed to determine the accuracy of problem joint reporting outside of Europe, where the term was developed and validated.

Despite presenting the highest amount of FVIII utilisation, infusion rates for participants in North America were similar to those in Australia and Europe/Middle East. The high median body weight of North American participants in this study compared to European/Middle Eastern participants may contribute to higher dosing per infusion, as dosing recommendations are weight based [3]; additionally, more frequent exclusive EHL use by participants in Australia vs North America (Figure S1) may explain the lower overall use of FVIII product, as switching from SHL to EHL prophylaxis reduces injection frequency [32].

Participants at sites in regions known to have limited access to FVIII products and thus lower utilisation—Africa, South America, and East Asia [26]—had worse Haemo-QoL-A outcomes than the ones in Australia, Europe/Middle East, and North America. For participants in both South America and Africa, Treatment Concern (e.g., “I worry about the availability of haemophilia products”) had lower domain scores than other regions, reflective of the well-recognised issues in those regions [26,28,30,31,33]. For participants in Africa, Consequences of Bleed (e.g., “I worry about accidents”) was the most impacted domain, possibly due to limited FVIII supply and a lack of haemophilia management education [30,31,34]. Overall, the observed differences between participants at trial sites grouped by region likely resulted from many confounding factors, including FVIII treatment regimens, type of FVIII used, access to treatment, and regional differences in education about haemophilia [35,36,37].

Overall, safety outcomes were consistent with other studies of individuals with severe HA [18,38,39]. AEs not related to HA observed during the on-study period were as expected for an otherwise healthy, young adult male population. The relatively high incidence of some AEs (such as arthropathy) reflects the underlying disease burden of HA.

Potential study limitations include visits scheduled at irregular intervals following the usual standard of care/routine clinical practice for the study site and the collection of QoL measures at a single time point. The population enrolled in this study was very heterogeneous in terms of age, race, medical history, and access to medical treatment, and it represents a specific cohort that is potentially interested in pursuing gene therapy for HA. This may have resulted in selection bias, as illustrated by the AAV5+ individuals who chose not to enrol after screening. Participants seeking better outcomes than those provided by their current FVIII prophylaxis may have been particularly motivated to enrol. Additionally, the mandated 6 months of high-quality retrospective data may have biased enrolment towards well-informed participants compliant with treatment. Site selection may also have been biased, as participating sites were chosen based on capability for gene therapy trials. Future studies that evaluate current prophylaxis therapies and clinical outcomes in developing countries are recommended to more fully characterize the state of haemophilia care worldwide.

## 5. Conclusions

Despite high prophylaxis adherence, the continued occurrence of spontaneous and joint bleeding events requiring treatment and impaired physical functioning was evident in our study. These results illustrate real-world shortcomings associated with regular FVIII prophylaxis for this cohort of individuals with severe HA, for whom additional haemostatic options are needed.

## Figures and Tables

**Table 1:** Patient demographics and baseline characteristics.

Parameter	Australia (*n* = 27)	Europe/ Middle East ^a^ (*n* = 95)	South America ^b^ (*n* = 54)	East Asia ^c^ (*n* = 32)	North America ^d^ (*n* = 48)	Africa ^e^ (*n* = 38)	Overall Enrolled Population (*N* = 294)
Age at enrolment, median (min, max) years	31.0 (18.0, 71.0)	33.0 (18.0, 70.0)	27.0 (18.0, 47.0)	40.0 (20.0, 66.0)	32.0 (19.0, 70.0)	28.0 (18.0, 67.0)	31.0 (18.0, 71.0)
Male sex, *n* (%)	27 (100.0)	95 (100.0)	54 (100.0)	32 (100.0)	48 (100.0)	38 (100.0)	294 (100.0)
Race, *n* (%)							
Asian	2 (7.4)	6 (6.3)	0	32 (100.0)	2 (4.2)	0	42 (14.3)
Black or African American	0	1 (1.1)	10 (18.5)	0	5 (10.4)	14 (36.8)	30 (10.2)
White	24 (88.9)	65 (68.4)	44 (81.5)	0	39 (81.3)	13 (34.2)	185 (62.9)
Other	1 (3.7)	0	0	0	0	0	1 (0.3)
Not provided ^f^	0	23 (24.2)	0	0	2 (4.2)	11 (28.9)	36 (12.2)
Hispanic or Latino ethnicity	1 (3.7)	2 (2.1)	0	0	1 (2.1)	0	4 (1.4)
Weight, mean (SD), kg	83.3 (18.1)	77.4 (14.9)	78.9 (20.4)	61.8 (11.0)	84.5 (19.6)	67.9 (18.7)	79.0 (18.1)
History of hepatitis B ^g^, *n* (%)	3 (11.1)	20 (21.1)	1 (1.9)	8 (25.0)	7 (14.6)	5 (13.2)	44 (15.0)
History of hepatitis C ^g^, *n* (%)	12 (44.4)	43 (45.3)	12 (22.2)	20 (62.5)	24 (50.0)	7 (18.4)	118 (40.1)
History of HIV, *n* (%)	0	4 (4.2)	0	0	6 (12.5)	0	10 (3.4)
Participants with problem joints ^h^, *n* (%)	9 (33.3)	42 (44.2)	5 (9.3)	18 (56.3)	20 (41.7)	7 (18.4)	101 (34.4)
Number of problem joints ^h^, *n* (%)							
0	18 (66.7)	53 (55.8)	49 (90.7)	14 (43.8)	28 (58.3)	31 (81.6)	193 (65.6)
1	3 (11.1)	14 (14.7)	5 (9.3)	11 (34.4)	6 (12.5)	6 (15.8)	45 (15.3)
2	3 (11.1)	9 (9.5)	0	5 (15.6)	3 (6.3)	1 (2.6)	21 (7.1)
3	2 (7.4)	8 (8.4)	0	2 (6.3)	4 (8.3)	0	16 (5.4)
>3	1 (3.7)	11 (11.6)	0	0	7 (14.6)	0	19 (6.5)

^a^ Belgium, Germany, Spain, France, UK, Israel, and Italy. ^b^ Brazil. ^c^ Korea and Taiwan. ^d^ US. ^e^ South Africa. ^f^ Due to patient privacy rules. ^g^ Includes cleared or cured infections. ^h^ Problem joints were identified by investigators at baseline and were defined as joints with any of the following symptoms: chronic joint pain, chronic synovitis, haemophilic arthropathy, limited motion, or recurrent bleeding. HIV, human immunodeficiency virus; SD, standard deviation.

**Table 2:** Pre-baseline and on-study annualised bleeding rates of the 6-Month Analysis Population.

	Pre-Baseline *n* = 225	On-Study *n* = 224	Total Duration *n* = 224
Annualised bleed rate, no. of bleeds/year			
All bleeds			
Mean (SD)	5.34 (10.1)	4.81 (6.83)	5.04 (7.53)
Median (range)	2.00 (0.0, 94.0)	2.05 (0.0, 37.8)	2.61 (0.0, 62.7)
Treated bleeds			
Mean (SD)	5.03 (9.35)	4.33 (6.39)	4.64 (7.00)
Median (range)	2.00 (0.0, 86.0)	1.85 (0.0, 37.8)	2.27 (0.0, 57.8)
Treated spontaneous bleeds			
Mean (SD)	2.98 (6.02)	1.93 (3.81)	2.35 (4.14)
Median (range)	0.00 (0.0, 58.0)	0.00 (0.0, 25.2)	0.94 (0.0, 39.3)
Treated traumatic bleeds			
Mean (SD)	2.05 (6.81)	2.40 (4.58)	2.29 (5.02)
Median (range)	0.00 (0.0, 86.0)	0.00 (0.0, 31.2)	0.88 (0.0, 55.9)
Treated joint bleeds			
Mean (SD)	3.74 (7.67)	2.74 (4.68)	3.19 (5.41)
Median (range)	2.00 (0.0, 72.0)	1.21 (0.0, 25.5)	1.44 (0.0, 47.0)
Treated problem joint bleeds			
Mean (SD)	1.22 (3.69)	0.66 (2.22)	0.91 (2.63)
Median (range)	0.00 (0.0, 32.0)	0.00 (0.0, 20.0)	0.00 (0.0, 21.4)

SD, standard deviation.

**Table 3:** Pre-baseline and on-study annualised FVIII utilisation and infusion rates of the 6-Month Analysis Population.

	Pre-Baseline	On-Study	Total Duration
Mean (SD) annualised FVIII utilisation rate, IU/kg/year			
*n*	223	222	222
All uses	3937.9 (1799.7)	3927.7 (1768.1)	3928.5 (1698.2)
Subtypes of usage			
Bleeds	261.9 (489.0)	186.7 (287.4)	219.2 (330.7)
Surgery/procedures	67.1 (411.4)	57.5 (202.7)	62.6 (222.1)
One-time prophylaxis ^a^	32.9 (154.6)	19.2 (68.9)	25.5 (82.0)
Usual prophylaxis ^b^	3575.9 (1756.9)	3664.3 (1685.4)	3621.2 (1637.8)
Mean (SD) annualised FVIII infusion rate, no. of infusions/year			
*n*	225	224	224
All uses	130.5 (45.2)	131.5 (48.5)	131.1 (45.2)
Subtypes of usage			
Bleeds	8.19 (15.6)	5.99 (8.91)	6.94 (10.3)
Surgery/procedures	1.75 (9.78)	1.72 (7.12)	1.77 (6.15)
One-time prophylaxis ^a^	0.96 (4.61)	0.51 (1.82)	0.71 (2.34)
Usual prophylaxis ^b^	119.6 (46.8)	123.3 (48.5)	121.6 (46.3)

^a^ Refers to a single infusion in anticipation for patients at high bleeding risk (e.g., before playing sports). ^b^ Refers to continuous or ongoing use aimed at maintaining FVIII activity above a certain target level. FVIII, Factor VIII; SD, standard deviation.

## Data Availability

De-identified individual participant data underlying these results will be made available together with the clinical protocol and data dictionaries, for non-commercial, academic purposes. Additional supporting documents may be available upon request. Investigators will be able to request access to these data and supporting documents via the Publication Data Request page at www.BioMarin.com (accessed on 14 December 2021) beginning 6 months and ending 2 years after publication. Data associated with any ongoing development program will be made available within 6 months after approval of the relevant product. Requests must include a research proposal clarifying how the data will be used, including the proposed analysis methodology. Research proposals will be evaluated relative to publicly available criteria available at www.BioMarin.com (accessed on 14 December 2021) to determine if access will be given, contingent upon the execution of a data access agreement with BioMarin Pharmaceutical Inc.

## Supplementary Materials

The following are available online at https://www.mdpi.com/article/10.3390/jcm10245959/s1, supplementary S1: Participating Sites and Investigators; supplementary S2: Methods; Figure S1: Participant FVIII prophylaxis product use by region in the 6-Month Analysis Population (*n* = 225); Table S1: Pre-baseline and on-study annualised treated bleeding rates of the 6-Month Analysis Population by region; Table S2: pre-baseline and on-study annualised FVIII utilisation rates of the 6-Month Analysis Population by region; Table S3: Pre-baseline and on-study annualised FVIII infusion rates of the 6-Month Analysis Population by region; Table S4: Overall incidence of AEs.
