# Supplementary material for: Real-World Rates of Bleeding, Factor VIII Use, and Quality of Life in Individuals with Severe Haemophilia A Receiving Prophylaxis in a Prospective, Noninterventional Study
Source: J Clin Med. 2021 Dec 18;10(24):5959. doi: 10.3390/jcm10245959 (PMC8705574; doi:10.3390/jcm10245959)
Supplement: Supplementary file 1 [file jcm-10-05959-s001.zip › jcm-1484315-supplementary.pdf]

SUPPLEMENTARY INFORMATION

This appendix has been provided by the authors to give readers additional information about their work.

Title: Real-world rates of bleeding, factor VIII use, and quality of life in individuals with severe haemophilia A receiving prophylaxis in a prospective, noninterventional study

Gili Kenet,[1] Yeu-Chin Chen,[2] Gillian Lowe,[3] Charles Percy,[3] Huyen Tran,[4] Annette von Drygalski,[5] Marc Trossaërt,[6] Mark Reding,[7] Johannes Oldenburg,[8] Maria Eva Mingot-Castellano,[9] Young Shil Park,[10] Flora Peyvandi,[11,12] Margareth C Ozelo,[13] Johnny Mahlangu,[14] Jennifer Quinn,[15] Mei Huang,[16] Divya B Reddy,[16] Benjamin Kim[16]

[1]The National Hemophilia Center, and Amalia Biron Research Institute of Thrombosis and Hemostasis, Sheba Medical Center, Tel Hashomer, Tel Aviv University, Tel Aviv, Israel; [2]Haemophilia Care and Research Center, Tri-Service General Hospital, National Defense Medical Center, Taipei, Taiwan; [3]West Midlands Comprehensive Care Haemophilia Centre, Queen Elizabeth Hospital, Birmingham, UK; [4]Haemostasis & Thrombosis Unit, Haemophilia Treatment Centre, The Alfred Hospital, Melbourne, Australia; [5]Department of Molecular Medicine, Scripps Research Institute, La Jolla, CA, USA; [6]Centre Régional de Traitement des Hémophiles, CHU de Nantes, Nantes, France; [7]Center for Bleeding and Clotting Disorders, University of Minnesota, Minneapolis, MN, USA; [8]Institute of Experimental Haematology and Transfusion Medicine and Center for Rare Diseases, University Hospital Bonn, Bonn, Germany; [9]Regional University Hospital, Carlos Haya, Málaga, Spain; [10]Kyung Hee University Hospital at Gangdong, Seoul, South Korea; [11]Fondazione IRCCS Ca' Granda Ospedale Maggiore Policlinico, Angelo Bianchi

Bonomi Hemophilia and Thrombosis Center and Fondazione Luigi Villa, Milan, Italy; [12]Università degli Studi di Milano, Department of Pathophysiology and Transplantation, Milan, Italy; [13]Hemocentro UNICAMP, Department of Internal Medicine, School of Medical Sciences, University of Campinas, Campinas, SP, Brazil; [14]Hemophilia Comprehensive Care Center, Charlotte Maxeke Johannesburg Academic Hospital, University of the Witwatersrand and NHLS, Johannesburg, South Africa; [15]BioMarin Pharmaceutical UK Ltd, London, UK; [16]BioMarin Pharmaceutical Inc., Novato, CA, USA

supplementary S1 Participating Sites and Investigators

Australia. Royal Adelaide Hospital: Chee Wee Tan (form. McRae); Royal Brisbane & Women's Hospital: Jane Mason; Royal Prince Alfred Hospital: Teh-Liane Khoo; The Canberra Hospital: Michael Pidcock; Fiona Stanley Hospital: Dominic Pepperell; The Alfred Hospital: Huyen Tran.

Belgium. Antwerp University Hospital: Alain Gadisseur; University Hospitals Leuven: Kathelijne Peerlinck; Cliniques Universitaires Saint Luc: Cedric Hermans.

Brazil. Hemocentro UNICAMP, University of Campinas: Margareth Ozelo.

France. Assistance Publique des Hopitaux de Marseille: Hervé Chambost; Hotel Dieu Hospital, Department of Clinical Hematology: Marc Trossaërt; Morvan Hospital: Brigitte Pan-Petesch; Regional University Hospital of Lille (CHRU of Lille): Antoine Rauch (form. Susen); Cochin Hospital: Natalie Stieltjes; Bicetre Hospital: Roseline D'Oiron.

Germany. Vivantes Hospital in Friedrichshain: Robert Klamroth; University Clinic Bonn: Johannes Oldenburg.

Israel. Chaim Sheba Medical Center: Gili Kenet.

Italy. Angelo Bianchi Bonomi Hemophilia and Thrombosis Centre: Flora Peyvandi; University Hospital Careggi: Giancarlo Castaman.

South Africa. Charlotte Maxeke Johannesburg Academic Hospital: Johnny Mahlangu; Groote Schuur Hospital: Cecile du Toit.

South Korea. Kyung Hee University Hospital: Young Shil Park; Kyungpook National University Hospital: Joon-Ho Moon; Ulsan University Hospital: Sang Kyu Park.

Spain. Complexo Hospitalario Universitario a Coruna: Maria Fernanda Lopez Fernandez; University Hospital Virgen del Rocio (HUVR), Hematology Pediatrics: Ramiro Nunez; Hospital Regional Universitario de Málaga: Eva Mingot-Castellano.

Taiwan. National Taiwan University Hospital: Sheng-Chieh Chou; Changhua Christian Children's Hospital: Ming-Ching Shen; Kaohsiung Medical University Hospital: Shyh-Shin Chiou; Taichung Veterans General Hospital: Jiaan-Der Wang; Service General Hospital: Yeu-Chin Chen.

United Kingdom. Hammersmith Hospital: Michael Laffan; Queen Elizabeth Hospital Birmingham: Gillian Lowe, Charles Percy; Addenbrooke's Hospital, Haemophilia and Thrombophilia Center: Emily Symington; Centre for Haemostasis and Thrombosis: Bella Madan; NHS Glasgow and Clyde: Catherine Bagot (form. Tait); Barts and The London School of Medicine and Dentistry: John Pasi; University Hospital Southampton NHS Foundation Trust: Sara Boyce; Royal Cornwall Hospitals NHS Trust: Michael Desmond Creagh; Churchill Hospital: Susan Shapiro.

United States. Vanderbilt Children's Hospital: Allison Wheeler; University of California San Diego School of Medicine: Annette Von Drygalski; Ann & Robert H. Lurie Children's Hospital of Chicago: Alexis Thompson; Nationwide Children's Hospital: Amy Dunn; University of California San Francisco: Andrew Leavitt; Wayne State University: Michael Callaghan; Washington University: Elaine Majerus; University of Florida: Anita Rajasekhar; Michigan State University: Roshni Kulkarni; Orthopaedic Hemophilia Treatment Center: Doris Quon; Indiana Hemophilia and Thrombosis Center: Amy Shapiro; Tulane University Hematology & Medical Oncology: Cindy Leissinger; Blood Research Institute at Blood Center of Wisconsin: Lynn Malec; University of Minnesota Division of Hematology Oncology and Transplant: Mark Reding, Marshall Mazepa; University of Colorado AMC: Michael Wang; St. Joseph's Children's Hospital: Erin Cockrell.

supplementary S2 Supplementary Methods

Exclusion criteria

1. Significant liver dysfunction with any of the following abnormal laboratory results:

- Alanine aminotransferase (ALT) > 1.25 × upper limit of normal (ULN)
- Aspartate aminotransferase (AST) > 1.25 × ULN
- Gamma glutamyl-transferase (GGT) > 1.25 × ULN
- Total bilirubin > 1.25 × ULN
- Alkaline phosphatase > 1.25 × ULN
- International normalised ratio (INR) ≥1.4

Participants whose liver laboratory assessments fall outside of these ranges may undergo repeat testing of the entire liver test panel within the same screening window and, if eligibility criteria are met on retest, may be enrolled after confirmation by the medical monitor.

2. Prior liver biopsy showing significant fibrosis of 3 or 4 as rated on a scale of 0–4 on the Batts-Ludwig or METAVIR scoring systems, or an equivalent grade of fibrosis if an alternative scale is used [1,2].

3. Liver cirrhosis of any aetiology as assessed by prior liver ultrasound.

4. Chronic or active hepatitis B as evidenced by positive serology testing (hepatitis B surface antigen [HBsAg], hepatitis B surface antibody [HBsAb], and hepatitis B core antibody [HBcAb]) and confirmatory HBV DNA testing.

5. Active hepatitis C, as evidenced by detectable hepatitis C virus RNA or currently on antiviral therapy.

6. Human immunodeficiency virus infection.

7. Active malignancy, except nonmelanoma skin cancer.

8. History of hepatic malignancy.

9. Evidence of any bleeding disorder not related to haemophilia A.

10. Has a medical condition or extenuating circumstance that, in the opinion of the investigator, might compromise the participant's ability to comply with protocol requirements, the participant's well-being or safety, or the interpretability of the participant's clinical data.

11. Prior treatment with any vector or gene transfer agent, or any medication prohibited by the protocol.

12. Concurrent enrolment in another clinical study unless it is an observational (noninterventional) clinical study that does not interfere with the requirements of the current protocol and by prior consultation with the medical monitor.

13. Unwilling to receive blood or blood products for treatment of an adverse event and/or a bleeding episode.

Haemophilia A disease measures

Bleeding episodes/Factor VIII replacement therapy

Participants must have high-quality documented historical data available concerning previous bleeding episodes and exogenous FVIII usage over the previous 6 months in order to be eligible to enrol in the study. During the screening period and prior to enrolment, the medical monitor will review each screened participant's prior bleeding episode and haemophilia medication logs to determine if they are of "high-quality." Elements that will be assessed to judge the quality of such historical data may include, but are not limited to, the following:

- Date, type (eg, joint, muscle, soft tissue, and other), location of bleeding episodes
- Date, name, dose (calculated in IU/kg), and reason for use (eg, usual prophylaxis, one-time prophylaxis, treatment for bleed, surgery) of haemophilia medications.

During the study, participants were asked on a weekly basis to report the use of FVIII replacement therapy and the number and type of bleeding episodes since the previous week by using the participant's diary provided by the sponsor.

Haemo-QoL-A

The Haemo-QoL-A questionnaire is a validated, haemophilia-specific, health-related quality of life questionnaire for adults [3]. It consists of 41 questions covering 6 domains (Physical Functioning, Role Functioning, Worry, Consequences of Bleeding, Emotional Impact and Treatment Concerns). Items are answered on a 6-point Likert-type scale, ranging from 0 (none of the time) to 5 (all the time). Higher scores mean better health-related QoL or less impairment for a particular subscale [4].

EQ-5D-5L

The EQ-5D-5L instrument is a self-reported questionnaire designed to measure general health status [5,6]. The EQ-5D-5L is composed of 2 parts: a descriptive system that assesses 5 levels of perceived problems (mobility, self-care, usual activities, pain/discomfort, and anxiety/depression) in 5 dimensions and the EQ Visual Analog Scale (EQ VAS) assessment for overall health. The score ranges from 0 (poor health) to 1 (perfect health). The second section is a 20 cm VAS [7]. The recorded measurement on this line produces a score in which 100 is best and 0 is the worst health state [7].

Haemophilia Activities List

The Haemophilia Activities List (HAL) measures the impact of haemophilia on self-perceived functional abilities in adults [8]. The instrument consists of multiple domains, including

lying/sitting/kneeling/standing, leg and arm function, use of transportation, self-care, household tasks, and leisure activities in which participants are asked to rate their level of difficulty with activities of daily living on a 6-point Likert-type scale from 1 (Impossible) to 6 (Never). For some items, participants are given the choice to answer, "Not applicable." HAL score is rated from 0 to 100, with 100 indicating no perceived functional difficulties [7].

Work Productivity and Activity Impairment plus Classroom Impairment Questions: Haemophilia-Specific

The Work Productivity and Activity Impairment plus Classroom Impairment Questions: Haemophilia-Specific (WPAI+CIQ:HS) instrument is designed to measure the effect of disease symptom severity on work productivity and classroom productivity (if applicable) [9]. The WPAI+CIQ:HS questionnaire yields scores related to work/classroom absenteeism, reduced on-the-job effectiveness, overall work/classroom impairment, and activity impairment. WPAI+CIQ:HS outcomes are expressed as impairment percentages, with higher numbers indicating greater impairment and less productivity [10].

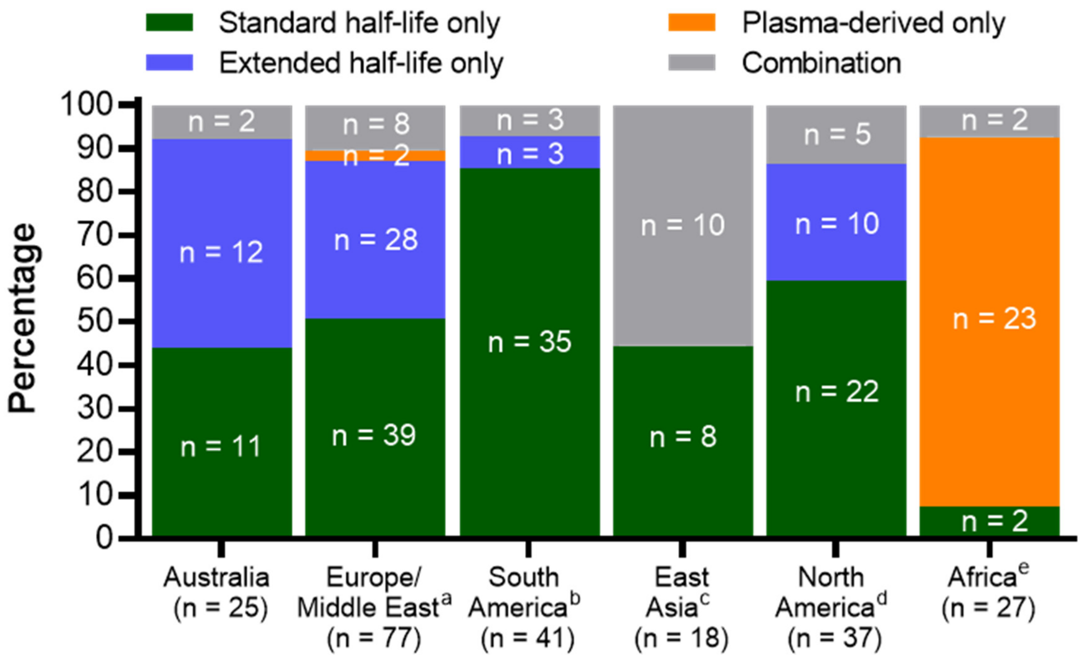

Figure S1. Participant FVIII prophylaxis product use by region in the 6-Month Analysis Population (n = 225). Combination includes participants who switched FVIII product types during the study period. [a]Belgium, Germany, Spain, France, UK, Israel, and Italy. [b]Brazil. [c]Korea and Taiwan. [d]US. [e]South Africa. FVIII, factor VIII.

Table S1. Pre-baseline and on-study annualised treated bleeding rates of the 6-Month Analysis Population by region

	Pre-baseline	On-study	Total duration
**Annualised bleed rate, no. of bleeds/year**			
Australia	n = 25	n = 25	n = 25
Mean (SD)	0.88 (1.42)	2.30 (2.29)	1.69 (1.55)
Median (range)	0.00 (0.0, 4.0)	1.68 (0.0, 7.0)	1.67 (0.0, 5.9)
Europe/Middle East[a]	n = 77	n = 76	n = 76
Mean (SD)	5.27 (7.30)	4.89 (5.99)	4.97 (5.85)
Median (range)	2.00 (0.0, 36.0)	2.83 (0.0, 33.3)	2.31 (0.0, 34.3)
South America[b]	n = 41	n = 41	n = 41
Mean (SD)	2.44 (3.83)	2.41 (4.61)	2.42 (4.05)
Median (range)	0.00 (0.0, 14.0)	0.00 (0.0, 23.8)	0.80 (0.0, 19.3)
East Asia[c]	n = 18	n = 18	n = 18
Mean (SD)	14.78 (24.47)	4.94 (8.35)	9.75 (16.08)
Median (range)	5.00 (0.0, 86.0)	1.78 (0.0, 30.8)	4.62 (0.0, 57.8)
North America[d]	n = 37	n = 37	n = 37
Mean (SD)	2.86 (5.18)	4.26 (6.97)	3.70 (5.56)
Median (range)	0.00 (0.0, 26.0)	1.79 (0.0, 37.8)	1.83 (0.0, 25.3)
Africa[e]	n = 27	n = 27	n = 27
Mean (SD)	8.59 (5.68)	7.25 (8.73)	7.71 (6.02)
Median (range)	8.00 (0.0, 22.0)	4.25 (0.0, 31.2)	5.85 (0.0, 26.0)

[a]Belgium, Germany, Spain, France, UK, Israel, and Italy. [b]Brazil. [c]Korea and Taiwan. [d]US. [e]South Africa.

SD, standard deviation.

Table S2. Pre-baseline and on-study annualised FVIII utilisation rates of the 6-Month Analysis Population by region

	Pre-baseline	On-study	Total duration
**Mean (SD) annualised FVIII utilisation rate, IU/kg/year**			
Australia			
Overall (n = 24)	3879 (927)	3876 (955)	3878 (923)
Standard half-life only (n = 10)	3424 (1108)	3580 (1220)	3513 (1159)
Extended half-life only (n = 12)	4218 (680)	4039 (710)	4117 (672)
Plasma-derived only (n = 0)	NA	NA	NA
Combination of products[a] (n = 2)	4119 (264)	4376 (578)	4270 (454)
Europe/Middle East[b]			
Overall (n = 77)	3974 (1710)	3963 (1576)[g]	3972 (1463)[g]
Standard half-life only (n = 39)	3929 (1228)	4129 (1344)	4048 (1258)
Extended half-life only (n = 28)	4016 (2242)	3559 (1427)[h]	3765 (1643)[h]
Plasma-derived only (n = 2)	4094 (1257)	7941 (3782)	6371 (1745)
Combination of products[a] (n = 8)	3894 (1873)	3524 (1289)	3656 (1333)
South America[c]			
Overall (n = 41)	3325 (1526)	3457 (1612)	3396 (1546)
Standard half-life only (n = 35)	3265 (1225)	3391 (1434)	3335 (1307)
Extended half-life only (n = 3)	5925 (2299)	5795 (2234)	5851 (2262)
Plasma-derived only (n = 0)	NA	NA	NA
Combination of products[a] (n = 3)	1421 (370)	1888 (269)	1663 (78.7)
East Asia[d]			
Overall (n = 18)	3557 (1093)	3599 (829)	3576 (835)
Standard half-life only (n = 8)	2806 (775)	3211 (1026)	3023 (782)
Extended half-life only (n = 0)	NA	NA	NA
Plasma-derived only (n = 0)	NA	NA	NA
Combination of products[a] (n = 10)	4158 (942)	3910 (487)	4019 (594)
North America[e]			
Overall (n = 36)	5613 (2292)	5386 (2513)	5456 (2351)

Standard half-life only (n = 22)	5625 (2044)	5263 (2102)	5376 (1987)
Extended half-life only (n = 9)	5313 (797)	5114 (944)	5204 (797)
Plasma-derived only (n = 0)	NA	NA	NA
Combination of products[a] (n = 5)	6100 (4719)	6418 (5355)	6259 (5040)
Africa[f]			
Overall (n = 27)	2838 (1034)	2864 (1054)	2857 (1022)
Standard half-life only (n = 2)	1901 (721)	2057 (18.6)	2000 (290)
Extended half-life only (n = 0)	NA	NA	NA
Plasma-derived only (n = 23)	2934 (1061)	2967 (1105)	2956 (1064)
Combination of products[a] (n = 2)	2667 (677)	2489 (444)	2576 (557)

[a]Includes participants who switched FVIII product types during the study period. [b]Belgium, Germany, Spain, France, UK, Israel, and Italy. [c]Brazil. [d]Korea and Taiwan. [e]US. [f]South Africa. [g]n = 76. [h]n = 27.

FVIII, Factor VIII; NA, not applicable; SD, standard deviation.

Table S3. Pre-baseline and on-study annualised FVIII infusion rates of the 6-Month Analysis Population by region

	Pre-baseline	On-study	Total duration
**Mean (SD) annualised FVIII infusion rate, no. of infusions/year**			
Australia			
Overall (n = 25)	118 (34.3)	119 (39.2)	119 (36.6)
Standard half-life FVIII only (n = 11)	147 (28.3)	150 (33.2)	149 (30.7)
Extended half-life FVIII only (n = 12)	94.5 (18.4)	90.2 (20.3)	92.1 (18.9)
Plasma-derived FVIII only (n = 0)	NA	NA	NA
Combination of FVIII products[a] (n = 2)	102 (2.83)	120 (25.0)	113 (16.2)
Europe/Middle East[b]			
Overall (n = 77)	126 (37.4)	125 (42.0)[g]	126 (37.8)[g]
Standard half-life FVIII only (n = 39)	136 (38.8)	141 (43.1)	139 (40.4)
Extended half-life FVIII only (n = 28)	113 (34.1)	105 (30.1)[h]	109 (28.8)[h]
Plasma-derived FVIII only (n = 2)	125 (24.0)	188 (53.5)	162 (22.3)
Combination of FVIII products[a] (n = 8)	118 (35.5)	106 (22.9)	111 (25.1)
South America[c]			
Overall (n = 41)	163 (60.0)	172 (63.1)	168 (60.2)
Standard half-life FVIII only (n = 35)	170 (60.5)	177 (61.8)	174 (60.3)
Extended half-life FVIII only (n = 3)	102 (19.1)	100 (19.4)	101 (19.3)
Plasma-derived FVIII only (n = 0)	NA	NA	NA
Combination of FVIII products[a] (n = 3)	140 (42.1)	185 (77.1)	163 (54.3)
East Asia[d]			
Overall (n = 18)	108 (34.0)	105 (33.7)	106 (27.0)
Standard half-life FVIII only (n = 8)	89.8 (21.4)	106 (46.6)	98.7 (31.1)
Extended half-life FVIII only (n = 0)	NA	NA	NA
Plasma-derived FVIII only (n = 0)	NA	NA	NA
Combination of FVIII products[a] (n = 10)	123 (36.0)	104 (21.3)	112 (23.0)
North America[e]			
Overall (n = 37)	135 (44.4)	126 (42.7)	129 (41.0)

Standard half-life FVIII only (n = 22)	145 (37.1)	131 (36.2)	136 (32.5)
Extended half-life FVIII only (n = 10)	105 (12.0)	103 (11.4)	104 (8.30)
Plasma-derived FVIII only (n = 0)	NA	NA	NA
Combination of FVIII products[a] (n = 5)	148 (84.8)	151 (83.5)	150 (84.2)
Africa[f]			
Overall (n = 27)	115 (30.6)	124 (29.3)	120 (29.2)
Standard half-life FVIII only (n = 2)	89.0 (15.6)	114 (1.44)	104 (5.16)
Extended half-life FVIII only (n = 0)	NA	NA	NA
Plasma-derived FVIII only (n = 23)	120 (30.1)	127 (30.6)	124 (29.8)
Combination of FVIII products[a] (n = 2)	90.0 (31.1)	101 (17.3)	95.5 (24.0)

[a]Includes participants who switched FVIII product types during the study period. [b]Belgium, Germany, Spain, France, UK, Israel, and Italy. [c]Brazil. [d]Korea and Taiwan. [e]US. [f]South Africa. [g]n = 76. [h]n = 27.

FVIII, Factor VIII; NA, not applicable; SD, standard deviation.

Table S4. Overall incidence of AEs

AE category, n (%)	Enrolled population (N = 294)
Any AE	128 (43.5)
Any SAE[a]	14 (4.8)
Any AE of CTCAE Grade ≥3	15 (5.1)
AEs reported in ≥1% of participants overall	
Nasopharyngitis	18 (6.1)
Arthralgia	17 (5.8)
Upper respiratory tract infection	11 (3.7)
Headache	10 (3.4)
Cough	6 (2.0)
Haemophilic arthropathy	6 (2.0)
Back pain	5 (1.7)
Oropharyngeal pain	5 (1.7)
Musculoskeletal pain	4 (1.4)
Anxiety	3 (1.0)
Constipation	3 (1.0)
Face injury	3 (1.0)
Pyrexia	3 (1.0)
Syncope	3 (1.0)
Viral infection	3 (1.0)

[a]CTCAE Grade 3 SAEs (n = 12; 4.1%) included one each for diverticulitis, perineal abscess, staphylococcal bacteraemia, haemorrhoidal haemorrhage, oesophageal haemorrhage, haemophilic arthropathy, macular fibrosis, acute cholecystitis, syncope, haematuria, renal colic, and haematoma; no Grade 4 or 5 SAEs were reported.

AE, adverse event; CTCAE, Common Terminology Critera for Adverse Events; SAE, serious AE.

REFERENCES

1. Batts KP, Ludwig J. Chronic hepatitis. An update on terminology and reporting. *Am J Surg Pathol.* 1995;19(12):1409-1417.

2. Bedossa P, Poynard T. An algorithm for the grading of activity in chronic hepatitis C. The METAVIR Cooperative Study Group. *Hepatology.* 1996;24(2):289-293.

3. Rentz A, Flood E, Altisent C, et al. Cross-cultural development and psychometric evaluation of a patient-reported health-related quality of life questionnaire for adults with haemophilia. *Haemophilia.* 2008;14(5):1023-1034.

4. Haemo-QoL Study Group. Scoring Manual. http://haemoqol.de/scoring/manual/. Accessed December 15, 2020.

5. EuroQol Group. EuroQol--a new facility for the measurement of health-related quality of life. *Health Policy.* 1990;16(3):199-208.

6. Brooks R. EuroQol: the current state of play. *Health Policy.* 1996;37(1):53-72.

7. Taylor S, Room J, Barker K. Physical activity levels in men with Haemophilia-A single centre UK survey. *Haemophilia.* 2020;26(4):718-725.

8. van Genderen FR, Westers P, Heijnen L, et al. Measuring patients' perceptions on their functional abilities: validation of the Haemophilia Activities List. *Haemophilia.* 2006;12(1):36-46.

9. Recht M, Neufeld EJ, Sharma VR, et al. Impact of acute bleeding on daily activities of patients with congenital hemophilia with inhibitors and their caregivers and families: observations from the Dosing Observational Study in Hemophilia (DOSE). *Value Health.* 2014;17(6):744-748.

10. Reilly Associates. WPAI Scoring. http://www.reillyassociates.net/WPAI_Scoring.html; Accessed December 15, 2020. 2002.
